# Headache in Workers: A Matched Case–Control Study

**DOI:** 10.3390/ejihpe12120130

**Published:** 2022-12-07

**Authors:** Reparata Rosa Di Prinzio, Gabriele Arnesano, Igor Meraglia, Nicola Magnavita

**Affiliations:** 1 Health Systems and Service Research, Post-Graduate School of Health Economics and Management, Facoltà di Medicina e Chirurgia, Università Cattolica del Sacro Cuore, 00168 Rome, Italy; 2 Occupational Medicine Unit, Bambino Gesù Children’s Hospital IRCCS, 00165 Rome, Italy; 3 Post-Graduate School of Occupational Health, Department of Life Sciences and Public Health, Facoltà di Medicina e Chirurgia, Università Cattolica del Sacro Cuore, 00168 Rome, Italy; 4 Department of Science of Woman, Child and Public Health, Fondazione Policlinico Universitario Agostino Gemelli IRCCS, 00168 Rome, Italy

**Keywords:** anxiety, depression, sleep, aggression, diet, cholesterol, blood pressure, glycemia, stress, lifestyle

## Abstract

A case–control study including 446 workers reporting headaches (cases; 136 males and 310 females, mean age 46.71 ± 10.84 years) and 446 age- and sex-matched colleagues without headaches (controls; mean age 45.44 ± 10.13) was conducted in the second half of 2020 in a sample drawn from socio health and commercial services companies to investigate the association of headache with lifestyle, metabolic, and work-related factors. Workers suffering from headache reported higher body weight (OR: 1.92, 95% CI: 1.46–2.53, *p* < 0.001), higher blood cholesterol (OR: 2.01, 95% CI: 1.46–2.77, *p* < 0.001), triglyceride (OR: 2.01, 95% CI: 1.20–3.35, *p* < 0.01), blood glucose (OR: 1.91, 95% CI: 1.16–3.24, *p* < 0.01), and blood pressure levels (OR: 1.76, 95% CI: 1.23–2.52, *p* < 0.01). In the year preceding the survey, cases had experienced a higher frequency of workplace violence (OR: 2.29, 95% CI: 1.25–4.20, *p* < 0.01 for physical aggression, OR: 2.22, 95% CI: 1.45–3.41, *p* < 0.001 for threat, OR: 2.74, 95% CI: 1.72–4.38, *p* < 0.001 for harassment) and were more frequently distressed (effort/reward ratio > 1) (OR: 1.82, 95% CI: 1.39–2.40, *p* < 0.001) than the controls. Compared to the controls, cases also had higher scores on anxiety and depression scales, lower scores on happiness, and lower levels of sleep quality (*p* < 0.001). The association of headaches with metabolic and mental health problems suggests that monitoring headaches in the workplace could help to identify workers at risk of impairment.

## 1. Introduction

Headache is a common problem, affecting up to 90% of individuals during their lifetime [1], that has a considerable impact on both individuals and society [2]. Studies have highlighted an increasing rate of specialty referrals, inappropriate treatment, and advanced imaging for simple headache. Managing headaches is still a challenge for primary care physicians and may result in inappropriate primary care management [3]. Headache disorders are the third cause of years lived with disability globally but rank second among young adults aged 15–49 years and first among young women [4]. Disability due to headaches (especially migraine) not only leads to a considerable reduction in the quality of life for affected individuals [5], but also produces an economic burden on society on account of long-term decreased productivity resulting from headache-related absenteeism and presenteeism [6,7,8]; United States estimates suggest that 16% of presenteeism is attributable to this problem [9]. Empirical studies have shown that effective treatment of headache is expected to recover a significant rate of lost productivity, resulting in a cost-saving intervention [10].

Headache has a very wide variety of possible causative factors and associated comorbidities, including metabolic factors such as hypertension [11,12], obesity [13,14,15], dyslipidemia [16], and hyperglycemia [17]. Lifestyle [18], sleep behavior [19,20], and anxiety and depression [21,22] are also associated with headache. Occupational stress [23,24,25] and psychosocial factors present in the workplace, such as low skill discretion, low decision authority, role conflicts, bullying, and effort–reward imbalance, could be among the triggers of headache [26]. There are reciprocal relationships between these factors. Indeed, according to a literature review, burnout, which is a consequence of unmanaged occupational stress, is a significant predictor of headaches, metabolic changes, insomnia, and job dissatisfaction [27]. Sleep disturbances and headache disorders share common brain structures and pathogenic mechanisms and often occur together: headache can promote sleep disturbances, and sleep disturbances can also precede or trigger a headache attack [28]. Identifying, through a case–control study, the factors associated with headaches in people who have not yet entered a diagnostic or therapeutic path can offer the doctor who collects the first symptom report an opportunity to initiate the necessary investigations.

In the literature, there are many studies that have evaluated patients with headache using a case–control design. Most of these studies relied on patients from hospitals or migraine centers [29,30,31,32,33,34]. Other studies looked at national or regional databases to derive data regarding work activity. For example, employees with chronic headaches and without chronic disease were selected from The Netherlands Working Conditions Survey conducted in 2013. The comparison demonstrated that job demand and job resources are important for work ability in employees with chronic headaches. Furthermore, results suggest that these employees benefit more strongly from supervisor support than employees without chronic headache [35]. A study using the 1996 to 1999 Medical Expenditure Panel Survey data on individuals with migraine headache showed that, contrary to expectations, a higher level of access to care was significantly associated with an increased likelihood of missing work and with missing a greater number of workdays [36]. All these studies referred to people who already had a definite diagnosis and had therefore undergone treatments, but to the best of our knowledge, we have not found any studies on untreated adults.

However, headache is a very common symptom, and it would be interesting to know if those who suffer from headaches but have not undertaken a diagnostic/therapeutic path to control the disease have been exposed to occupational or metabolic risks differently from their colleagues who do not have a headache. The identification of factors associated with the appearance of this symptom in workers may be useful to guide screening and treatment of headache-related diseases in the workplace and thereby reduce the impact of these disturbances on productivity. If predictors of headache can be identified, it may be possible to develop a guideline to enable health and safety services to manage this disorder more adequately. This study aimed to verify which occupational, metabolic, and psychological factors emerge from the comparison between workers with and without headaches, to stimulate further research on the role of these factors as determinants of the different forms of headache, and on the disorders that could be associated with recurrent headaches.

## 2. Materials and Methods

### 2.1. Participants

In Italy, workers who are exposed to professional risks undergo regular medical examinations in the workplace. This case–control study was conducted between 5 June 2020 and 31 December 2020 in companies in the social health and commercial services sectors. We followed the STROBE guidelines for case–control studies [37] (Table S1).

The eligibility criterion was the presence of headaches in people exposed to occupational risks for at least one year. Workers were asked to answer the question: “Do you suffer from headaches?” Workers who answered “yes” were defined as “cases”. Since the study was aimed at workers and not patients, we excluded all subjects who were being treated for migraines or other forms of headache. We also excluded workers who had suffered head injuries, road accidents in the past year or who were professionally exposed to chemicals that can cause headaches (carbon monoxide, nitrogen oxide). Among all the subjects undergoing medical examination in the same company, workers of the same sex and similar age (±4 years old) who were not suffering from headache were invited to participate as controls (1:1 matching). We limited matching criteria to only two strong confounders and recruited cases and controls from the same work environment to reduce the potential risk of selection bias.

### 2.2. Procedure

The size of the sample was evaluated according to Epitools [38], making the hypothesis of an expected proportion of 0.05 exposed in the controls, an assumed odds ratio equal to 2.1, and a level of confidence equal to 0.95 and 0.80 of power. The calculation gave a minimal number of 439 participants for each group for the detection of a significant difference between the two groups.

The research was conducted in accordance with the Helsinki Declaration. The study received the approval of the Ethics Committee of the Università Cattolica del Sacro Cuore (3008, 5 June 2020). Each participant expressed informed consent and self-completed a questionnaire during the medical examination.

### 2.3. Instruments

To analyze differences between the two groups, four major areas of headache determinants were explored, including lifestyle factors, metabolic and mental health, and work-related factors.

Study lifestyle factors (diet, physical exercise, smoking habits, and alcohol consumption) were classified using single-item 4-point scales. Categorical variables were dichotomously divided, to compare non-smokers with smokers or former smokers; active subjects (carrying out physical exercise at least three times per week) with non-active subjects (failing to do so); and controlled diet (subjects who reduced sugar, fat, and salt consumption every day) with uncontrolled diet (subjects who failed to control their diet).

The workers were also questioned about metabolic health and possible treatment for arterial hypertension, hypercholesterolemia, hypertriglyceridemia, hyperglycemia, and overweight/obesity. Answers to these questions were validated using data from blood tests, medical history, and anthropometric measurements reported in each subject’s health record. Cut-off levels were defined according to the International Diabetes Federation (IDF) [39], the National Cholesterol Education Program Expert Panel on Detection, Evaluation, and Treatment of High Cholesterol in Adults (NCEP/ATPIII) [40], and the American Association of Clinical Endocrinologists (AACE) [41]. Metabolic parameters were then classified as either “normal” or “high or undergoing treatment”.

We considered sleep, anxiety, depression, and happiness as mental health measures. Sleep quality was studied using the Italian version [42] of the “Pittsburgh Sleep Quality Index” (PSQI) [43], consisting of 18 issues forming 7 components, each of which had a score ranging from 0 to 3. The minimum score is zero, and the maximum is 21. Higher scores indicate worse sleep quality. A score of 5 or more indicates a bad sleeper. The response referred to the previous month. The reliability of PSQI has been verified using Cronbach’s alpha [44] (0.83 [45]).

Anxiety and depression were assessed using the Italian version [46] of the “Goldberg’s Anxiety and Depression Scale” (GADS) [47], referring to the previous 10-day period. The GADS is composed of two scales of 9 binary questions each; one point is awarded for each positive answer. A score of 5 or more on the anxiety subscale, or 2 or more on the depression subscale, indicates suspected clinically evident anxiety or depression [47]. Cronbach’s alphas in this study showed internal consistency reliability (0.85 for the anxiety sub-scale and 0.79 for the depression sub-scale).

Happiness was measured using the Abdel-Khalek single item (“Do you feel happy in general?”) answered on an 11-point scale (0–10) [48]. Work-related factors concerning violence and occupational injuries that had occurred in the previous year were evaluated with binomial (yes/no) questions on physical aggression, threat, and harassment. Finally, we took into account work-related factors. Work-related distress was measured using the Italian version [49] of Siegrist’s short “Effort–Reward Imbalance” (ERI) scale [50], containing three questions for the effort variable and seven for the reward variable. All items had graded responses on a 4-point Likert scale from 1 = totally agree to 4 = totally disagree, so the resulting sub-scales were between 3 and 12 (effort) and between 7 and 28 (reward), respectively. The weighted effort/reward imbalance ratio indicates the level of occupational stress. According to the literature [51], we classified values as “normal” or “high” using a cut-off of 1. Cronbach’s alphas were 0.74 for the effort sub-scale and 0.79 for the reward sub-scale [50].

Exposure to workplace violence in the year prior to the visit was investigated through three questions from the Arnetz’s Violent Incident Form (VIF) [52]. These questions were (1) “Over the past 12 months, you happened to be the victim of a physical assault while you were working? (For physical assault means an attack, with or without weapons, that could cause or not cause physical damage)”; (2) “Over the past 12 months, you happened to be a threat while you were working? (A threat refers to the intention of causing physical damage)”; (3) “Over the past 12 months, you happened to be harassed while you were working? (Harassment is any act, words, attitudes, actions, annoying or unpleasant, which creates a hostile work environment)”. The performance of night work was recorded with a single question: “Do you do night work?”. All these questions provide binary answers (yes or no).

### 2.4. Data Analysis

Statistical analyses were performed using χ^2^ tests; a *p* value < 0.05 was considered statistically significant. Frequencies in the case and control groups were compared by chi-square test. Using univariate logistic regression, the odds ratio (OR) and 95% confidence interval (95% CI) of the workers with headaches, compared with the controls, were calculated. Continuous variables obtained from the questionnaires (effort, reward, ERI, happiness, anxiety, depression, PSQI score) were tested for normality by applying statistical tests (Kolmogorov–Smirnov and Shapiro–Wilks). As their distribution was non-normal (Table S2), they were compared using the Mann–Whitney U test. Only questionnaires with complete data sets were considered in the analyses; those with missing data were no longer accepted.

The IBM Corp. Released 2019. IBM SPSS Statistics for Windows, Version 26.0. Armonk, NY, USA: IBM Corp was used for the analyses.

## 3. Results

A total of 446 cases and 446 controls participated in the survey. None of the workers submitted to medical examination shunned the question: “Do you suffer from headaches?” which served to identify cases and potential controls. None of the cases refused to fill in the questionnaire used in the survey. Five potential cases were excluded because they had suffered traffic accidents or were being treated at headache centers. Four of the potential controls screened for age and sex by the same companies refused to participate. The reason given for not participating was a lack of time to complete the questionnaire.

Of the 892 participants, two-thirds consisted of females; this proportion reflects the composition of the productive sectors concerned, where female labor is prevalent. Demographic characteristics of the sample are shown in Table 1.

The reliability analyses of psychometric scales were measured by Cronbach’s alpha statistic to verify that the questionnaire used in the survey has the same characteristics as the original tool. In this study, Cronbach’s alphas indicated high consistency (0.893 for PSQI, 0.852 for the GADS anxiety sub-scale, 0.794 for the GADS depression sub-scale, 0.860 for the ERI effort sub-scale, and 0.701 for the ERI reward sub-scale).

No significant disparity was found for current diet control, physical exercise, smoking, and alcohol habits between the two groups. However, metabolic changes were significantly more prevalent in the cases than in the controls. Univariate logistic regression revealed that cases had a higher frequency of high cholesterol blood levels (OR: 2.01, 95% CI: 1.46–2.77, *p* < 0.001), triglyceride blood levels (OR: 2.01, 95% CI: 1.20–3.35, *p* < 0.01), overweight or obesity (OR: 1.92, 95% CI: 1.46–2.53, *p* < 0.001), hyperglycemia (OR: 1.91, 95% CI: 1.16–3. Exposure to workplace violence was reported more frequently in the cases than in the controls. Using univariate logistic regression, the odds ratio of all types of violence was more elevated in the workers with headaches (OR: 2.29, 95% CI: 1.25–4.20, *p* < 0.01 for physical aggression, OR: 2.22, 95% CI: 1.45–3.41, *p* < 0.001 for threat, OR: 2.74, 95% CI: 1.72–4.38, *p* < 0.001 for harassment, OR: 2.05, 95% CI: 1.26–3.33). No difference was observed in the occurrence of work injuries or night work between the two groups. Cases were more frequently in a condition of high stress (ERI > 1) than controls (OR: 1.82, 95% CI: 1.39–2.40, *p* < 0.001) (Table 2).

In the workers suffering from headaches, the median scores of anxiety, depression, and sleep problems were significantly higher than in the controls (*p* < 0.001). The level of happiness was significantly lower in cases than in the controls (*p* < 0.001) (Table 3). By constructing a multiple logistic regression model including stress, anxiety, depression, sleep quality, and happiness as predictors, anxiety was significantly associated with headache (Table S3).

## 4. Discussion

This study on active workers showed that metabolic, occupational, and psychological factors are significantly different between subjects with and without headaches. In particular, the workers with headaches suffered from hypertension, obesity, dyslipidemia, and hyperglycemia far more frequently than their colleagues not affected by headaches. The association between headache and components of the metabolic syndrome suggests that this common symptom may indicate an increased cardiovascular risk, at least in some cases. In fact, the relationship between migraine headache, which globally accounts for about a quarter of headache cases [53], and cardiovascular risk is documented in the literature. The duration, frequency, and severity of a migraine attack have all been related to cholesterol and triglyceride abnormalities [16,54]. Arterial hypertension is frequently associated with migraine, and this co-morbidity may be sustained by endothelial dysfunction [55]. The association of migraine with high blood glucose levels is in agreement with the “neuroenergetic” hypothesis, which suggests impaired brain glucose metabolism [56]. Obesity plays a role in migraine [57,58]. Although the results of individual studies are often conflicting, their meta-analysis has shown that migraine is associated with a 1.4-fold increase in the risk of cardiovascular and cerebrovascular events, myocardial infarction, and stroke [59]. Other studies have indicated an association between migraine and vascular diseases in regions other than the brain and the heart [60]. The pathogenetic mechanisms of these relationships are unknown, but it is hypothesized that some type of vascular dysfunction may be at the origin of both migraine and vascular risk [60]. A higher prevalence of metabolic syndrome was observed in people with migraine with aura in a stratified random sample of the general Belgian population [61]. A prospective cohort study of the general population in Norway observed that migraine with aura is associated with an increased risk of developing metabolic syndrome [62]. Although all these studies suggest that there is a relationship between migraine and metabolic syndrome, a systematic review concluded that studies, hitherto mainly conducted on female hospital outpatients, are not sufficient to evaluate the strength of the association and to produce a meta-analysis [63]. Furthermore, studies on hospital patients with migraine do not include all individuals with recurrent headaches that interfere with their work. Episodic migraine affects an estimated 12% of the population, and about 1–2% of the global population suffers from chronic migraine [64]. In occupational cohorts, over 40% of workers suffer from headaches, and one in five have a headache that significantly affects their working capacity [65]. In fact, in most workers, headache disorders are much more prevalent than migraine [66]. Furthermore, patients with metabolic syndrome have headaches other than migraines, with a headache subtype distribution similar to that of the general population [67]. As a result, because no epidemiological data on the association between headache disorders and cardiovascular risk are available other than those referring to migraine, we must assume, as a precautionary principle, that all types of headaches may be associated with metabolic risk factors and cardiovascular risk indicators. Therefore, in all patients who complain of headaches, the physician should look into metabolic factors.

In our observations, lifestyle (diet, tobacco and alcohol consumption, physical exercise) failed to reveal significant differences between the cases and controls. This finding, which seems to contradict the results of the literature, is explained by the characteristics of the sample, which was made up entirely of workers who undergo periodic medical surveillance and often operate in workplaces where smoking and alcohol are prohibited. These workers were already aware of their metabolic problems and, on the advice of their doctor, had taken appropriate countermeasures such as diet control, abolition of alcohol and smoking, and increased physical activity. The results of the study would probably have been different if we had investigated lifestyles adopted in the past rather than current habits. In fact, the association between diet and headache is well known [68]. Dietary factors may interact with migraine’s neurobiological pathogenic mechanisms [69]. Studies clearly demonstrate that triggers such as stress, menstrual cycle changes, weather changes, sleep disturbances, alcohol, and certain foods can induce multiple migraine attacks [70]. Some foods (e.g., chocolate, processed meats, monosodium glutamate, aspartame, fatty foods) act as common triggers for migraine attacks [71,72], while a ketogenic diet can decrease migraine occurrence by impacting on its dysfunctional mechanisms [68]. Alcohol consumption has also been reported to be significantly associated with migraine [18,73]. Furthermore, both daily smoking and environmental tobacco exposure in indoor spaces and workplaces have been associated with an exacerbation of headaches [18,74]. A healthy lifestyle that includes adequate sleep, stress management, and regular eating habits may help to prevent triggers and the development of chronic migraine over time [70]. Regular physical exercise has also been shown to be a non-pharmacological therapy for migraine [54,75,76,77].

The lack of association between headaches and night work could also be explained by selective factors that lead workers with recurrent headaches to avoid night work. Night shift work, in fact, has been associated with an increased incidence of unspecific headache as well as with medical diagnosis of migraine [78]. However, the limited number of studies and their conflicting results do not allow us to extrapolate definite conclusions about this relationship [79].

Our study demonstrated the existence of significant differences in occupational risk factors between cases and controls. All forms of physical or verbal violence experienced in the year preceding our survey were significantly more prevalent in the cases than in the controls. The number of cases of occupational distress (ERI > 1) was also greater among workers suffering from headaches than among their non-affected colleagues. The role of both factors is supported by data in the literature. In abused women, headaches are associated with the violence experienced [80]. In patients with migraine, there is often a history of abuse [81]. A recent study of nurses at a health company showed that violence suffered in the previous year was associated with the risk of headaches [82]. Data obtained for 50,205 respondents to the Fifth Korean Working Conditions Survey indicated that workers who had experienced violence were more likely to experience headache [83]. Longitudinal studies in the workplace found that bullying and harassment are predictors of headaches [84].

Numerous studies involving the workplace indicate that occupational stress or perceived injustices are associated with headaches in workers [26,85]. Headache is one of the commonest symptoms in workers with burnout (a consequence of chronic stress not effectively managed) [27]. Loneliness and poor social support, factors that in workers exposed to occupational stress determine a high-risk condition known as “isostrain” [86], are associated with headache [87]. Studies conducted on patients with chronic migraine have demonstrated that the latter is a vital factor in perceived stress, which affects the quality of life [88]. Psychosocial stress and excessive use of digital technology contribute to the so-called “21st century headache” [89]. High stress and sleep problems can predict headache severity in individuals with chronic headache [90].

As we saw in our sample, psychosocial factors play a leading role in headache disorders. Psychological factors play a significant role in frequency, intensity, and headache-related disability [91]. Genetic factors govern the relationship between social stressors and headache [92]. Tension headaches and migraines are linked to sleep problems [28]. Sleep problems, especially a lack of sleep and poor sleep quality, are often associated with headache [20,93,94,95]. Headache occurrence is associated with sleep problems and anxiety, as well as with depression [96]. Anxiety has been reported as one of the commonest comorbidities of migraine [97]. A bidirectional relationship between headache and depression has been hypothesized since headache represents one of the main symptoms in the onset of depression, and depression plays a role when migraine becomes chronic [98,99]. Migraine can play an important role in increasing the incidence of depression in affected patients [100].

Finally, we observed that the cases were less happy than the controls. In fact, it is well known that headache may negatively affect the quality of life [101]. Interestingly, an effective adaptation called “hedonic habituation” has been observed in high or low-frequency migraine sufferers [102], and weekend headaches may be seen as a negative expression of high work-related stress on days devoted to relaxation [103]. Job satisfaction, which is also related to decision control and control over the intensity of work, can impact on headache [104]. The perception of migraine intensity may interfere with levels of job-related gratification [105].

### 4.1. Limitations and Strengths

The limitations of our study are due to the case–control design, which was unable to determine a cause–effect relationship. Studies of this type have the sole function of documenting the associations but cannot indicate whether the associated factor is a cause or a consequence or whether there is reciprocity between the two variables. Furthermore, we adopted matching to improve study accuracy [106], even though it has been observed that this procedure may introduce selection bias [107]. To reduce this problem, according to recommendations [108], we have matched only for strong confounders, age, and sex, and we have recruited controls from the same work environment, thereby decreasing the likelihood of unknown confounders. Another limitation of the study was the use of a sample from social health and commercial companies, which limits the generalizability of the results to other work sectors.

One of the strong points of this study is that it considered a population of individuals engaged in their work rather than a series of patients treated in a clinic. This made it possible to study cases not yet diagnosed and treated, but inevitably prevented the researchers from distinguishing between the different types of headache disorders.

The simultaneous investigation of physical, behavioral, and mental factors as well as those related to the working environment was another important feature of our study. However, this was inevitably linked to the limitation of having to simplify the detection tools in order not to overburden the investigation. Consequently, some variable measurements were based on only one item, and this certainly reduced the strength of the study.

### 4.2. Practical Implications and Future Research

Our study indicates that such a common ailment as headache is significantly associated with metabolic and mental health problems. This should prompt doctors to actively investigate the phenomenon, even in those otherwise healthy patients who have never been diagnosed or treated in a headache clinic. Despite its widespread prevalence, migraine remains under-diagnosed and under-treated. Diagnosis and management of headache disorders should be conducted according to the most up-to-date guidelines, which derive from the consensus of scientific societies at an international level [109]. Education and reassurance, avoidance of triggers, and nonpharmacological treatments when appropriate can be usefully applied in the workplace.

Since headaches can have a considerable impact on daily life activities [65] and are responsible for a considerable economic burden at an occupational level, workplace-based treatment and stress prevention programs could constitute a financial and clinical investment [108,110,111]. Public health intervention could aim to promote behavioral changes that would modify risk factors. To improve life quality and expectancy globally, in addition to screening programs for headache, anxiety, depression, and sleep problems, it might be useful to set up policies that specifically address violence at work [112]. Furthermore, initiatives based on mindfulness techniques for the management of chronic pain [113] should be adopted in the workplace to prevent headache. Educational and physical exercise programs aimed at alleviating headache and neck symptoms [114], as well as behavioral or somatic manifestations [115], should be encouraged.

Although headaches are generally not a symptom directly caused by work, the doctor in charge of occupational health surveillance should actively seek this symptom to facilitate workers’ access to proper care.

## 5. Conclusions

This study showed that workers with headaches have a higher frequency of metabolic disorders and a worse level of mental health than those without headaches. They are also more exposed to workplace stress and violence than colleagues without headaches. Identifying these possible predictors or co-morbidities of headache can help health services to manage this disorder more adequately.

This study demonstrates how appropriate it is for the occupational doctor in charge of the health surveillance of workers to systematically investigate the presence of headaches, in consideration of the productive impact of this morbid condition. He/she can indicate to the worker the opportunity to carry out the most appropriate investigations to clarify the diagnosis, assess the presence of comorbidities, and establish the most appropriate treatment. He/she will also be able to explain to the company the opportunity to conduct screening campaigns in the workplace. The diffusion of the morbid phenomenon and the high prevalence of occupational factors that can favor it support the usefulness and economic benefit of health promotion campaigns.

Findings support the idea that monitoring headache in the workplace could help to identify workers at risk of impairment. Furthermore, it is reasonable to think that interventions on lifestyle, health, and occupational factors, according to the health promotion programs already started in many companies, could improve the life quality and performance of workers.

## Figures and Tables

**Table 1:** Demographic characteristics of cases and controls.

Variable	Cases	Controls
Sex		
Male—*n* (%)	136 (30.49)	136 (30.49)
Female—*n* (%)	310 (69.51)	310 (69.51)
Age—mean ± s.d. (years)	46.71 ± 10.84	45.44 ± 10.13

Note: s.d.: standard deviation.

**Table 2:** Association of potential predictive factors with headache (odds ratio in decreasing order).

Measured Area	Variables	Cases *n* (%)	Controls *n* (%)	Pearson χ^2^	OR (95% CI)
Lifestyle factors	Diet uncontrolled vs. controlled	351 (78.9) vs. 94 (21.1)	335 (75.3) vs. 110 (24.7)	1.63	1.23 (0.90–1.68)
	Physical exercise non-active vs. active	408 (80.3) vs. 100 (19.7)	372 (81.0) vs. 87 (19.0)	0.80	1.16 (0.84–1.61)
	Smoking habits yes vs. no	141 (31.6) vs. 305 (68.4)	144 (32.4) vs. 301 (67.6)	0.05	1.04 (0.78–1.37)
	Alcohol consumption yes vs. no	154 (34.8) vs. 289 (65.2)	157 (35.4) vs. 287 (64.6)	0.04	1.03 (0.78–1.35)
Metabolic factors	Cholesterol blood level “high/undergoing treatment” vs. “normal”	131 (29.4) vs. 314 (70.6)	76 (17.2) vs. 366 (82.8)	18.58 **	2.01 (1.46–2.77) **
	Triglyceride blood level “high/undergoing treatment” vs. “normal”	46 (10.3) vs. 399 (89.7)	24 (5.4) vs. 418 (94.6)	7.35 *	2.01 (1.20–3.35) *
	Weight “high/undergoing treatment” vs. “normal”	203 (45.5) vs. 243 (54.5)	135 (30.3) vs. 310 (69.7)	21.80 **	1.92 (1.46–2.53) **
	Glycemia “high/undergoing treatment” vs. “normal”	42 (9.4) vs. 404 (90.6)	23 (5.2) vs. 423 (94.8)	5.99 **	1.91 (1.16–3.24) **
	Blood pressure “high/undergoing treatment” vs. “normal”	93 (20.9) vs. 353 (79.1)	58 (13.0) vs. 388 (87.0)	9.77 *	1.76 (1.23–2.52) *
Work-related factors	Harassment yes vs. no	67 (15.0) vs. 379 (85.0)	27 (6.1) vs. 419 (93.9)	19.03 **	2.74 (1.72–4.38) **
	Physical aggression yes vs. no	35 (7.8) vs. 411 (92.2)	16 (3.6) vs. 430 (96.4)	7.51 *	2.29 (1.25–4.20) *
	Threat yes vs. no	71 (15.9) vs. 375 (84.1)	35 (7.8) vs. 411 (92.2)	13.88 **	2.22 (1.45–3.41) **
	Stress high vs. normal	204 (46.2) vs. 238 (53.8)	141 (32.0) vs. 300 (68.0)	18.65 **	1.82 (1.39–2.40) **
	Work injury yes vs. no	20 (4.5) vs. 426 (95.5)	17 (3.8) vs. 428 (96.2)	0.25	1.18 (0.61–2.29)
	Night work yes vs. no	102 (22.9) vs. 343 (77.1)	105 (23.6) vs. 339 (76.4)	0.07	0.96 (0.70–1.31)

Notes: 95%CI: confidence interval at 95%; OR: odds ratio; vs: versus. * *p* < 0.01, ** *p* < 0.001.

**Table 3:** Comparison of anxiety, depression, happiness, and sleep quality in cases and controls using the Mann–Whitney U test.

Variable (Score Range)	Cases		Controls		Mann–Whitney *p* Value
	Mean ± s.d.	Median (CI95%)	Mean ± s.d.	Median (CI95%)	
Anxiety (0–9)	4.90 ± 2.85	5 (3–7)	2.58 ± 2.62	2 (0–5)	<0.001
Depression (0–9)	3.15 ± 4.48	3 (1–5)	1.71 ± 1.97	1 (0–3)	<0.001
Sleep problems (0–21)	6.67 ± 3.66	6 (4–9)	4.50 ± 2.95	4 (2–6)	<0.001
Happiness (0–10)	6.34 ± 2.00	7 (5–8)	7.01 ± 1.99	7 (6–8)	<0.001

Note: s.d.: standard deviation.

## Data Availability

The data presented in this study are available on Zenodo. https://doi.org/10.5281/zenodo.7029734 (accessed on 1 December 2022).

## Supplementary Materials

The following supporting information can be downloaded at: https://www.mdpi.com/article/10.3390/ejihpe12120130/s1, Table S1: Checklist of items that should be included in reports of case–control studies according to STROBE guidelines. Table S2. Tests of normality (Kolmogorov-Smirnov with Lilliefors Significance Correction and Shapiro-Wilks) for continuous variables derived from questionnaires. Table S3. Multivariate logistic regression of factors associated with headache.
