# Supplementary material for: Headache in Workers: A Matched Case–Control Study
Source: Eur J Investig Health Psychol Educ. 2022 Dec 7;12(12):1852–66. doi: 10.3390/ejihpe12120130 (PMC9777382; doi:10.3390/ejihpe12120130)
Supplement: Supplementary file 1 [file ejihpe-12-00130-s001.zip › ejihpe-1912728-supplementary.pdf]

Table S1. Checklist of items that should be included in reports of case-control studies according to STROBE guidelines [27].

	Item No	Recommendation	Lines
**Title and abstract**	1	(*a*) Indicate the study's design with a commonly used term in the title or the abstract	2, 13
		(*b*) Provide in the abstract an informative and balanced summary of what was done and what was found	13-27
**Introduction**			
Background/rationale	2	Explain the scientific background and rationale for the investigation being reported	32-76
Objectives	3	State specific objectives, including any prespecified hypotheses	76-80
**Methods**			
Study design	4	Present key elements of study design early in the paper	83-98
Setting	5	Describe the setting, locations, and relevant dates, including periods of recruitment, exposure, follow-up, and data collection	84-85
Participants	6	(*a*) Give the eligibility criteria, and the sources and methods of case ascertainment and control selection. Give the rationale for the choice of cases and controls	87-98
		(*b*) For matched studies, give matching criteria and the number of controls per case	93-98
Variables	7	Clearly define all outcomes, exposures, predictors, potential confounders, and effect modifiers. Give diagnostic criteria, if applicable	109-163
Data sources/ measurement	8	For each variable of interest, give sources of data and details of methods of assessment (measurement). Describe comparability of assessment methods if there is more than one group	112-171
Bias	9	Describe any efforts to address potential sources of bias	96-98
Study size	10	Explain how the study size was arrived at	99-103
Quantitative variables	11	Explain how quantitative variables were handled in the analyses. If applicable, describe which groupings were chosen and why	112-171
Statistical methods	12	(*a*) Describe all statistical methods, including those used to control for confounding	165-173
		(*b*) Describe any methods used to examine subgroups and interactions	NA
		(*c*) Explain how missing data were addressed	170-171
		(*d*) If applicable, explain how matching of cases and controls was addressed	NA
		(*e*) Describe any sensitivity analyses	132-133, 139-140, 152-153
**Results**			
Participants	13	(a) Report numbers of individuals at each stage of study—eg numbers potentially eligible, examined for eligibility, confirmed eligible, included in the study, completing follow-up, and analysed	175-185

		(b) Give reasons for non-participation at each stage	175-185
		(c) Consider use of a flow diagram	NA
Descriptive data	14	(a) Give characteristics of study participants (eg demographic, clinical, social) and information on exposures and potential confounders	185
		(b) Indicate number of participants with missing data for each variable of interest	NA
Outcome data	15	Report numbers in each exposure category, or summary measures of exposure	NA
Main results	16	(*a*) Give unadjusted estimates and, if applicable, confounder-adjusted estimates and their precision (eg, 95% confidence interval). Make clear which confounders were adjusted for and why they were included	192-211
		(*b*) Report category boundaries when continuous variables were categorized	See item 11
		(*c*) If relevant, consider translating estimates of relative risk into absolute risk for a meaningful time period	NA
Other analyses	17	Report other analyses done—eg analyses of subgroups and interactions, and sensitivity analyses	187-191
**Discussion**			
Key results	18	Summarise key results with reference to study objectives	213-214
Limitations	19	Discuss limitations of the study, taking into account sources of potential bias or imprecision. Discuss both direction and magnitude of any potential bias	320-339
Interpretation	20	Give a cautious overall interpretation of results considering objectives, limitations, multiplicity of analyses, results from similar studies, and other relevant evidence	214-318
Generalisability	21	Discuss the generalisability (external validity) of the study results	341-374
**Other information**			
Funding	22	Give the source of funding and the role of the funders for the present study and, if applicable, for the original study on which the present article is based	NA

NA: not applicable.

Table S2. Tests of normality (Kolmogorov-Smirnov with Lilliefors Significance Correction and Shapiro-Wilks) for continuous variables derived from questionnaires.

	**Kolmogorov-Smirnov**			**Shapiro-Wilk**		
**Variable**	Statistic	df	*p*	Statistic	df	*p*
Effort	0.098	883	0.000	0.958	883	0.000
Reward	0.069	883	0.000	0.987	883	0.000
ERI	0.107	883	0.000	0.892	883	0.000
Anxiety	0.135	892	0.000	0.912	892	0.000
Depression	0.186	892	0.000	0.883	892	0.000
Happiness	0.176	859	0.000	0.935	859	0.000
PSQI	0.127	883	0.000	0.947	883	0.000

a. Lilliefors Significance Correction. df: degrees of freedom

Table S3. Multivariate logistic regression of factors associated with headache.

	p	OR	95% C.I. Lower	95% C.I. Upper
Sex (male)	0.339	1.174	0.845	1.631
Age	0.336	0.993	0.978	1.007
ERI	0.121	1.316	0.930	1.860
Anxiety	0.000	1.283	1.176	1.401
Depression	0.550	0.968	0.868	1.078
Happiness	0.787	1.012	0.928	1.104
PSQI_score	0.059	1.062	0.998	1.131
Constant	0.020	0.279		

OR= odds ratio; 95%CI confidence interval at 95%
